# Effects of UVC Irradiation on Growth and Apoptosis of* Scedosporium apiospermum* and* Lomentospora prolificans*

**DOI:** 10.1155/2018/3748594

**Published:** 2018-12-02

**Authors:** Watcharamat Muangkaew, San Suwanmanee, Pantira Singkum, Potjaman Pumeesat, Natthanej Luplertlop

**Affiliations:** Department of Microbiology and Immunology, Faculty of Tropical Medicine, Mahidol University, Bangkok, Thailand

## Abstract

*Scedosporium apiospermum *and* Lomentospora prolificans* are important fungal species isolated from immunocompromised patients. Previous studies have demonstrated that these filamentous fungi exist as saprophytes in the soil and showed the highest minimum inhibitory concentration to several drugs. We aimed to examine how UVC affects the* S. apiospermum* and* L. prolificans* by investigating the role of UVC on growth, induction of apoptosis by ethidium bromide (EB)/acridine orange (AO) staining, and transcriptomic study of caspase recruitment domain family, member 9 (CARD-9) gene. Our studies showed that 15 minutes of exposure to UVC light effectively increased reduction in both organisms and caused changes in colony morphology, color, and hyphal growth pattern. After 15 min of UVC irradiation, apoptotic cells were quantitated by EB/AO staining, and the percentage of apoptosis was 96.06% in* S. apiospermum* and 28.30% in* L. prolificans*. CARD-9 gene expression results confirmed that apoptosis was induced in* S. apiospermum* and* L. prolificans* after UVC treatment and that* S. apiospermum* showed a higher expression of apoptosis signaling than* L. prolificans*. Our study explored the effects of UVC in the inactivation of* S. apiospermum *and* L. prolificans*. We hope that our data is useful to other researchers in future studies.

## 1. Introduction

*Scedosporium apiospermum* species complex is a group of emerging opportunistic fungal pathogens increasingly found in immunocompromised patients [1].* S. apiospermum* and* Lomentospora prolificans* (former name* Scedosporium prolificans*) are medically important fungal species that were isolated from patients with* Scedosporium* infections [2–5]. Previous studies have demonstrated that these filamentous fungi exist as saprophytes in soil, particularly soil from industrial sites, urban playgrounds, agricultural fields, sewers, and polluted water, and are associated with organic matter [6, 7]. In Thailand, infections with* S. apiospermum* and* L. prolificans* have been reported [8, 9]. However, the clearance of* L. prolificans* is more complicated than* S. apiospermum* because it is more resistant to numerous conventional antifungal drugs [10].

Several studies have evaluated the use of ultraviolet (UV) light as a disinfectant [11, 12]. UV is divided into three wave bands, namely, UVA, UVB, and UVC. UVC (wavelength 100–280 nm) is highly germicidal and commonly used in sterilization [13]. Sullivan* et al*. determined the effects of UVC in prokaryotic and eukaryotic organisms [14] and showed that 99% of* Pseudomonas aeruginosa* and* Mycobacterium abscessus* were killed after 3–5 s of exposure and 99% of* Candida albicans* and* Aspergillus fumigatus* were killed after 15–30 s of exposure. Results of this study were supported by Dai* et al.* who investigated the effects of UVC light on* C*.* albicans* after an infection in a mouse with third-degree burns [15]. They demonstrated that UVC treatment carried out on day 0 and day 1 significantly reduced the fungal bioburden in the infected burns.

*S. apiospermum *and* L. prolificans* can be opportunistic infection and environmental contamination, especially the soil. Hence, the immunocompromised host could be susceptibility for infection [16]. So, these fungi possibly distribute and contaminate which can highly affect the immunocompromised host.

Studies have mainly focused on the role of UVC as a disinfectant, particularly its fungicidal effects. However, information on the role of UVC on the apoptosis pathway is lacking. The aim of this study was to examine how UVC affects* S. apiospermum *and* L. prolificans* by investigating the role of UVC on growth and induction of apoptosis.

## 2. Materials and Methods

### 2.1. Isolates and Culture Conditions

*S. apiospermum *CBS 117410 and* L. prolificans* CM324 were provided by Dr. Ana Alastruey Izquierdo (Servicio de Micología, Instituto de Salud Carlos III, Madrid, Spain). Each isolate was incubated on Sabouraud Dextrose Agar (SDA; Difco, USA) slants at 35°C for 7 days. Conidia were collected and suspended in phosphate-buffered saline (PBS, pH 7.4).

### 2.2. Qualitative Evaluation of UVC on the Growth of S.* apiospermum* and* L. prolificans*

To determine the effects of UVC on the growth of S.* apiospermum* and* L. prolificans*, 1 ml of the conidia suspension with a concentration of 1 × 10^6^ cells/ml was aliquoted into 6-well plates and exposed to UVC radiation of wavelength 254 nm (CL-1000 Ultraviolet Crosslinker, Canada) 54 mJ cm^−2^[13] for 15 min. Following that 20 *μ*l of suspension was transferred onto SDA plates and incubated at 37°C for 7 days. Colony morphology (diameter, color, and growth rate) was observed by the naked eye on each day until day 7. The remaining conidia suspension was centrifuged at 2,000 rpm for 5 min, and the pellet was resuspended in PBS (pH 7.4) and used apoptosis study by ethidium bromide (EB) and acridine orange (AO) staining, and transcriptomic study of the caspase recruitment domain family, member 9 (CARD-9) gene.

### 2.3. Scanning Electron Microscopy

The conidia suspension of* S. apiospermum* and* L. prolificans *after UVC exposure was transferred onto SDA plates and incubated at 37°C for 4 days; after that the fungal hyphae were collected and placed on a 13 mm circular coverslip. The cell morphology was determined by SEM. Firstly, the samples were fixed with 2.5% glutaraldehyde in sucrose phosphate buffer solution (one time for 1 h (.The fixative solution was aspirated out and the samples were dehydrated by methanol for 1 min and aspirated out until they were dry. After that, the samples were coated with gold and examined by a scanning electron microscope (model JSM-6610Lv, JEOL Ltd., Tokyo, Japan).

### 2.4. Apoptosis Study

Apoptotic cells were detected by staining with EB/AO as previously reported with minor modifications [17–19]. Briefly, 2 *µ*l of 100 *µ*g/ml EB and 100 *µ*g/ml AO each was added to 20 *µ*l of the sample, the and samples were immediately observed under a fluorescent microscope (Olympus/BX41). Live and apoptotic cells were determined. Live cells showed a green normal nucleus, whereas apoptotic cells showed condensed or fragmented chromatin.

### 2.5. Transcriptomic Study of the CARD-9 Gene

To investigate apoptosis at a molecular level, transcriptome levels of the apoptosis regulator gene CARD-9, the apoptosis related gene regulation, the expression of IL-1*β*, and also interaction with BCL10/CLAP were quantitated [20–22]. Total RNA from* S. apiospermum* CBS 117410 and* L. prolificans* CM324 after 15 min of UVC exposure and corresponding controls without UVC exposure were isolated using TRIzol® (Invitrogen, USA), according to the manufacturer's instructions. Approximately 1 × 10^6^ cells were lysed with 750 *μ*l of TRIzol® reagent, using chloroform as the separating phase. Isopropanol was used to precipitate RNA prior to washing with 75% ethanol and DNase treatment. RNase-free water was used to solubilize RNA. The RNA yield was determined with a NanoDrop 2000 spectrophotometer (Thermo Fisher, Wilmington, DE, USA) and the total RNA concentration was adjusted.

The change in CARD-9 expression after UVC exposure was determined using quantitative reverse transcriptase-polymerase chain reaction (qRT-PCR). RT-PCR was carried out using 80 ng/reaction of RNA template and PCR reaction mixture comprising KAPA SYBR® FAST One-Step qRT-PCR Master Mix (2X) kit (KAPA Biosystems, USA), 0.4 *μ*l of each 10 *μ*M forward and reverse primers (CARD-9: forward primer 5′-TCCGACCTGGAAGATGGCTCAC-3′, reverse primer 5′-CAGAGCTGCAAAGGGCTGTTTC-3′) [22], and 0.4 *μ*l of 50X RT Mix. PCR amplification was carried out in a CFX96 TouchTM Real-Time PCR Detection System (Biorad, Germany). A negative control that comprised all the reagents except RNA template was used. All the conditions were carried out in duplicate with the same RNA. The level of gene expression in the test and control samples was analyzed by qRT-PCR. *β*-Tubulin was used as a reference gene for qRT-PCR normalization using the following primers: *β*-tubulin forward 5 -GGTAACCAAATCGGTGCTGCTTTC-3′ and *β*-tubulin Reverse 5′-ACCCTCAGTGTAGTGACCCTTGGC-3′ [23]. RNA quantification was carried out according to the 2^ΔΔCt^ method. CARD-9 expression under UV irradiation was compared with the control conditions.

## 3. Results

### 3.1. Exposure Time Dependent Effect of UVC Radiation to* S. apiospermum* and* L. Prolificans*

**Figure 1:**
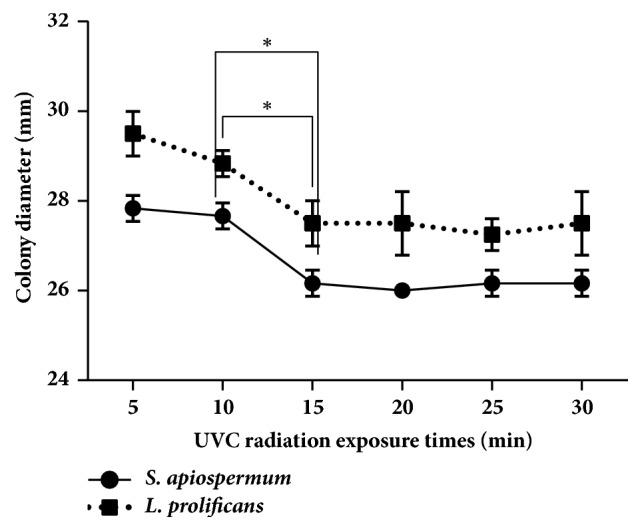
The UVC exposure time dependent of* S. apiospermum* CBS 117410 and* L. prolificans* CM324.

The initial test effect of UVC radiation: the time dependent effect was found to significantly decrease the size of colony which was found at 15-minute exposure, and after 15 min there was no significant decrease. Then we used 15-minute exposure in this study (Figure 1).

### 3.2. Effects of UVC on the Growth of* S. apiospermum* and* L. Prolificans*

**Figure 2:**
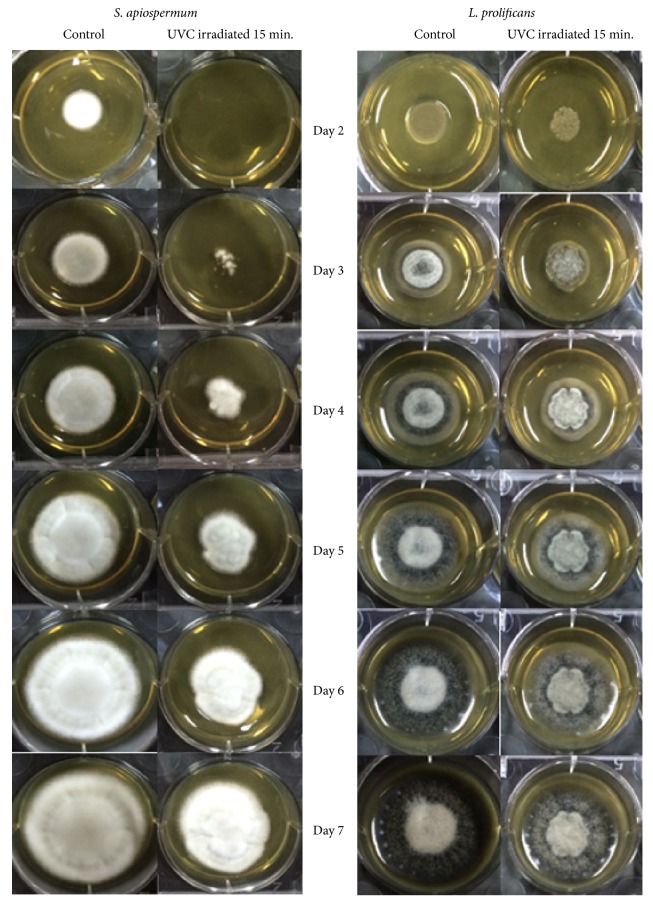
Colonies of* S. apiospermum* CBS 117410 and* L. prolificans* CM324 on SDA after 15 min of UVC exposure from days 2 to 7.

**Figure 3:**
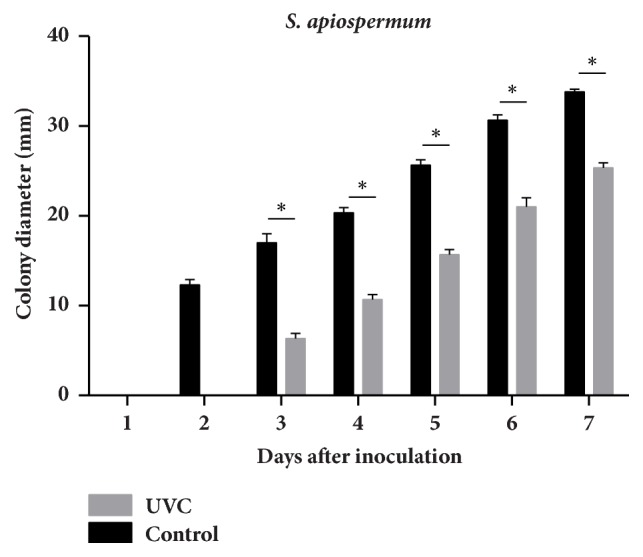
The growth kinetic of* S. apiospermum* CBS 117410 after 15 min of UVC exposure.

**Figure 4:**
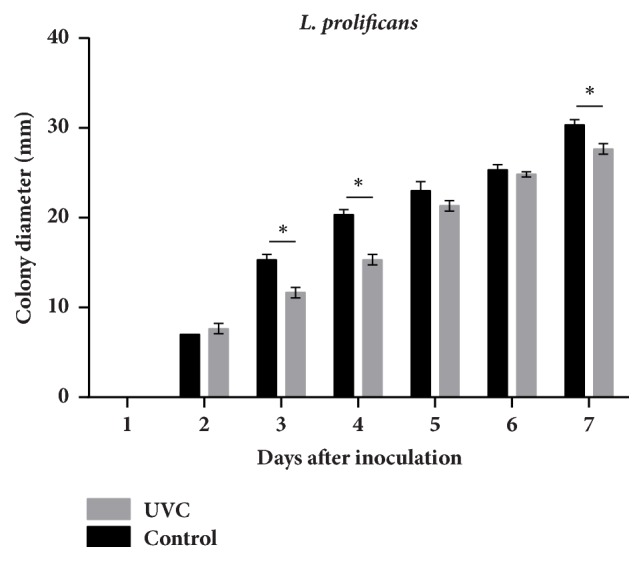
The growth kinetic of* L. prolificans* CM324after 15 min of UVC exposure.

The growth rate was determined by measuring the diameter of colonies and observing the morphology of colonies. Results showed that, in the controls (without UVC irradiation), the colony diameters of* S. apiospermum* and* L. prolificans* were significantly larger than those with UVC exposure (Figures 2–4). The edges of the colonies were circular and smooth in the controls of both* S. apiospermum* and* L. prolificans*. In contrast, the colonies of* S. apiospermum* and* L. prolificans* with UVC exposure had a serrated edge with a radiating halo; they were not circular in days 2–4, but gradually became circular in days 5–7. The color of* S. apiospermum *controls (without UVC irradiation) on SDA was white at day 2 before gradually turning gray (completely gray at day 7). The colonies of* S. apiospermum* with UVC exposure also showed the similar results, but with a pale shade of gray compared with the controls. The colonies of* L. prolificans* controls had black edges radiating from a center that showed a mix of black and gray hyphae, and colonies with UVC exposure were paler in color than the control.

### 3.3. Scanning Electron Microscopy(SEM)

**Figure 5:**
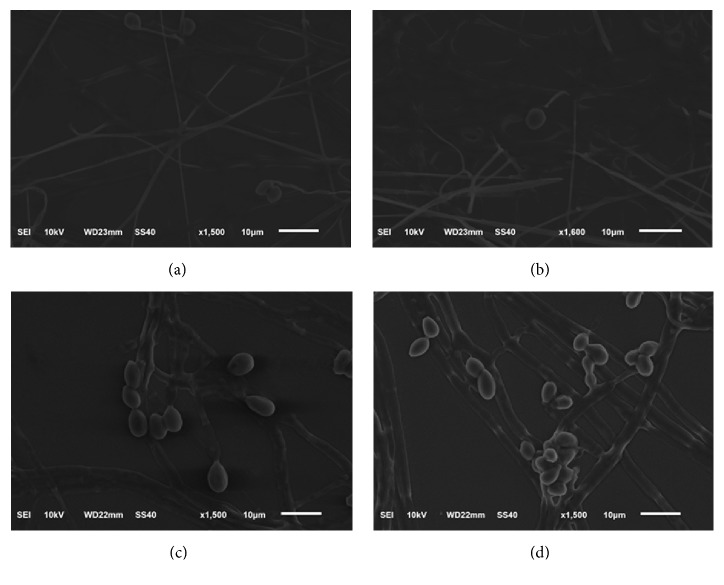
Cell morphology under SEM. (a)* S. apiospermum* CBS 117410 without UVC exposure; (b)* S. apiospermum* CBS 117410 after 15 min of UVC exposure; (c)* L. prolificans* CM324 without UVC exposure; and (d)* L. prolificans* CM324 after 15 min of UVC exposure.

Under SEM, there were no differences in the cell morphology of* S. apiospermum* and* L. prolificans*, with or without UVC exposure (Figure 5).

### 3.4. Effect of UVC on* S. apiospermum* and* L. prolificans* Apoptosis Pathways

**Figure 6:**
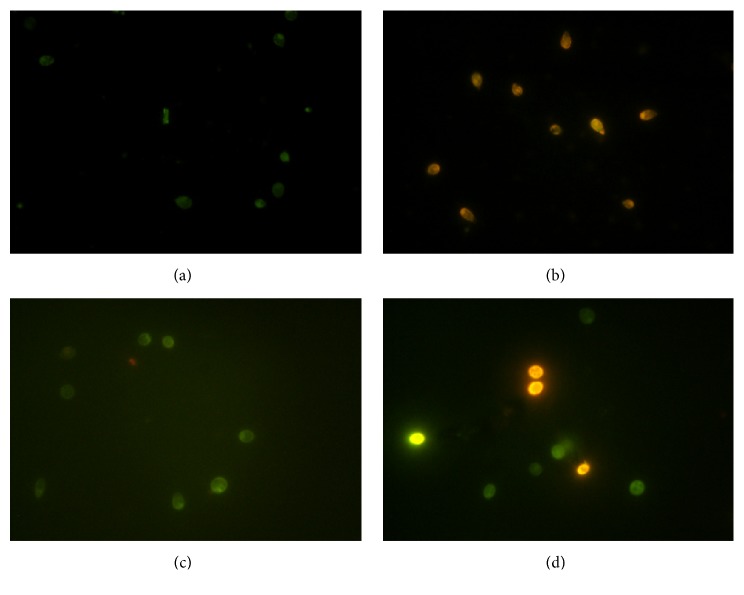
Apoptotic cells on ethidium bromide/acridine orange staining. Cells were observed using fluorescence microscopy. (a)* S. apiospermum* CBS 117410 without UVC exposure; (b)* S. apiospermum* CBS 117410 after 15 min of UVC exposure; (c)* L. prolificans* CM324 without UVC exposure; and (d)* L. prolificans* CM324 after 15 min of UVC exposure.

UVC induces apoptosis in several fungi [13, 24, 25]. In both* S. apiospermum* and* L. prolificans*, UVC exposure induced apoptosis, which was observed by chromatin condensation and orange staining of cells after EB and AO treatment (Figure 6). With UVC exposure, the percentage of apoptotic cells was 96.06% for* S. apiospermum* and 28.30% for* L. prolificans*. Without UVC exposure, the percentage of apoptotic cells was 2.38% for* S. apiospermum* and 2.04% for* L. prolificans*.

### 3.5. mRNA Expression of CARD-9 Gene

**Figure 7:**
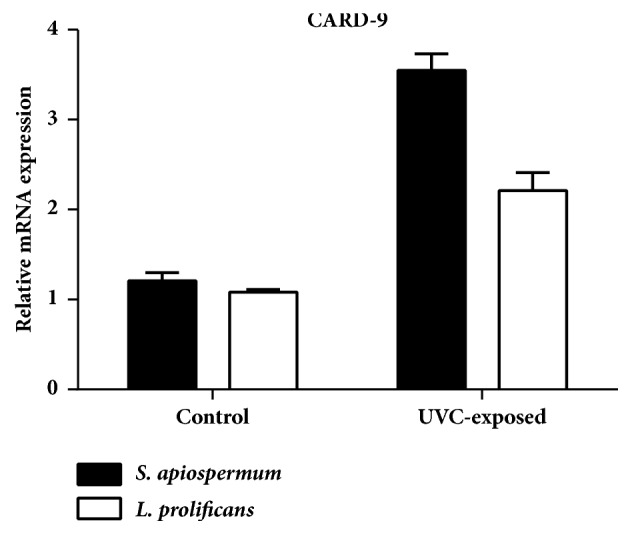
CARD-9 mRNA expression in* S. apiospermum* CBS 117410 and* L. prolificans* CM324 after 15 min of UVC exposure compared with controls. Gene expression was calculated using the 2^ΔΔCt^ method. Triplicate experiments were carried out to derive mean ± standard deviation. The CARD-9 expression levels were normalized to *β*-tubulin gene.

Increased CARD-9 mRNA expression was observed in both* S. apiospermum* and* L. prolificans* after 15 min of UVC exposure, compared with controls (without UVC irradiation). Interestingly,* S. apiospermum *showed a higher expression of apoptosis signaling than* L. prolificans *after UVC exposure, reaching statistical significance (P < 0.05) (Figure 7*).*

## 4. Discussion

*Scedosporium* species (including* L. prolificans*) are shown to be involved in opportunistic infections, particularly in immunocompromised patients. In Thailand,* S. apiospermum *has been reportedly found in brain abscesses of near-drowning and renal transplant patients [8, 26] and* L. prolificans *has been reportedly found in a case of disseminated infection in a patient with acute myeloid leukemia with prolonged febrile neutropenia (Damronglerd et al. 2014). In this patient,* L. prolificans* was shown to be resistant to antifungal agents such as amphotericin B, voriconazole, and posaconazole (minimal inhibitory concentration > 32 *μ*g/ml) [17]. In environmental investigation studies, we found that the* S. apiospermum* species complex is widespread in soils across Bangkok and detected predominance of* S. apiospermum *sensu stricto [27]. As mentioned above,* L. prolificans* showed high minimal inhibitory concentrations to many conventional antifungal drugs while* S. apiospermum *appeared to be more susceptible to these drugs [28]. Because of antifungal resistance, numerous studies have investigated new drugs in combination with conventional drugs for synergy in fungicidal activity [29–31].

Numerous studies have demonstrated the role of UV light as a disinfectant, particularly in bacteria. UV light is also used as a disinfectant in areas such as indoor swimming pools [32] and hospital surfaces [33]. UV light is divided into bands of UVA, UVB, and UVC. UVC light is commonly used as a tool for inactivation of microorganism. Lakretz* et al*. showed that UVC wavelengths between 254 nm and 270 nm were the most effective for inactivation of microorganisms, and wavelengths of 254, 260, or 270 nm were effective in biofilm control [34]. Therefore, the wavelength of 254 nm was selected in our study. To date, no study has explained the role of UVC irradiation on the growth of* S. apiospermum* and* L. prolificans*. In our study, we demonstrated the effectiveness of UVC radiation in reducing the growth of both organisms after 15 min of UVC exposure, which was accompanied by changes in the color and morphology of the colonies and hyphal growth pattern. In bacteria, UVC exerts its bactericidal activity through DNA damage [35]. Bak* et al.* showed that UVC killed* Staphylococcus aureus*,* Escherichia coli*, and* Pseudomonas aeruginosa* in a dose- and time-dependent manner, with no viable counts after 2 min of UVC exposure, while* C. albicans* was killed after 20 min of UVC exposure [36]. The microbicidal effects of UV irradiation were dependent on the genus and species of target microorganisms. Conner-Kerr* et al*. found that* Enterococcus faecalis* was more susceptible to killing by UV light than* S. aureus* [37] and UVC does not discriminate between antibiotic-resistant strains and susceptible strains [35, 37].

After conducting a review of literature, we were interested in UVC induced programmed cell death. In this study, we showed that apoptosis was induced in 96.06% of* S. apiospermum* and 28.30% of* L. prolificans *cells after UVC exposure. Our results show that the* S. apiospermum* was three times more sensitive to UVC than* L. prolificans*, adding value to the hypothesis that UVC acts is a genus/species dependent manner. We further explored the effects on apoptosis on a molecular level by analyzing the CARD-9 gene expression. CARD-9 can interact with the CARD domain of BCL10, a positive regulator of apoptosis and NF-*κ*B activation [38]. Gene expression results showed that CARD-9 expression was induced in* S. apiospermum* and* L. prolificans *cells after UVC treatment and* S. apiospermum* showed a higher expression of apoptosis signaling than* L. prolificans*. However, the morphological analysis through SEM did not show any change or significant point in UVC exposed; it might be from the UVC which did not affect directly morphology but affected the internal signal transduction involved in the growth of fungal and other functions which need to be further investigated.

A study by El-Azizi* et al*. demonstrated the effectiveness of UVC in combination with anti-staphylococcal antibiotics in the disinfection of catheter biofilms of methicillin-susceptible and methicillin-resistant staphylococcal strains [39]. Further studies could be carried out to ascertain the effectiveness of UVC light in combination with antifungal drugs on* S. apiospermum* and* L. prolificans*. Overall, our study observed the effects of UVC on inactivation of* S. apiospermum *and* L. prolificans*. Therefore, this knowledge could be applied for therapeutic approach such as UVC topical application of mycoses in burns, dermatophytes infection, and other superficial and subcutaneous fungal infections. However, further evaluations are required in terms of safety and efficacy. We hope that our data is useful to other researchers in future studies as well.

## Data Availability

The data used to support the findings of this study are available from the corresponding author upon request.
